# Methods for the field evaluation of quantitative G6PD diagnostics: a review

**DOI:** 10.1186/s12936-017-2017-3

**Published:** 2017-09-11

**Authors:** Benedikt Ley, Germana Bancone, Lorenz von Seidlein, Kamala Thriemer, Jack S. Richards, Gonzalo J. Domingo, Ric N. Price

**Affiliations:** 1 Global and Tropical Health Division, Menzies School of Health Research and Charles Darwin University, Darwin, Australia; 2 Shoklo Malaria Research Unit, Mahidol-Oxford Tropical Medicine Research Unit, Faculty of Tropical Medicine, Mahidol University, Mae Sot, Thailand; 3 Centre for Tropical Medicine and Global Health, Nuffield Department of Clinical Medicine, University of Oxford, Oxford, UK; 4 Mahidol-Oxford Tropical Medicine Research Unit (MORU), Bangkok, Thailand; 5 Malaria Elimination Program, Burnet Institute, Melbourne, VIC Australia; 6 Department of Medicine, University of Melbourne, Parkville, VIC Australia; 7 Victorian Infectious Diseases Service, Peter Doherty Institute for Infection and Immunity, Melbourne, VIC Australia; 8 Diagnostics Global Program, PATH, Seattle, WA USA

## Abstract

Individuals with glucose-6-phosphate dehydrogenase (G6PD) deficiency are at risk of severe haemolysis following the administration of 8-aminoquinoline compounds. Primaquine is the only widely available 8-aminoquinoline for the radical cure of *Plasmodium vivax*. Tafenoquine is under development with the potential to simplify treatment regimens, but point-of-care (PoC) tests will be needed to provide quantitative measurement of G6PD activity prior to its administration. There is currently a lack of appropriate G6PD PoC tests, but a number of new tests are in development and are likely to enter the market in the coming years. As these are implemented, they will need to be validated in field studies. This article outlines the technical details for the field evaluation of novel quantitative G6PD diagnostics such as sample handling, reference testing and statistical analysis. Field evaluation is based on the comparison of paired samples, including one sample tested by the new assay at point of care and one sample tested by the gold-standard reference method, UV spectrophotometry in an established laboratory. Samples can be collected as capillary or venous blood; the existing literature suggests that potential differences in capillary or venous blood are unlikely to affect results substantially. The collection and storage of samples is critical to ensure preservation of enzyme activity, it is recommended that samples are stored at 4 °C and testing occurs within 4 days of collection. Test results can be visually presented as scatter plot, Bland–Altman plot, and a histogram of the G6PD activity distribution of the study population. Calculating the adjusted male median allows categorizing results according to G6PD activity to calculate standard performance indicators and to perform receiver operating characteristic (ROC) analysis.

## Background

Glucose-6-phosphate dehydrogenase (G6PD) is an essential enzyme in the pentose phosphate pathway (PPP), the only pathway for human red blood cells (RBC) to maintain the cells’ redox potential by reducing NADP+ to NADPH [1, 2]. The enzyme consists of two dimers encoded by a gene on the long arm of the X chromosome. Non-synonymous mutations in the gene can decrease enzyme activity or reduce the stability of the enzyme, resulting in different degrees of G6PD deficiency. Because the gene is on the X chromosome, males are hemizygous and are either classified as G6PD deficient or normal by phenotype. Females have two gene copies which are expressed alternately in RBCs. Females therefore can be homozygous deficient (two gene copies with a deleterious mutation), heterozygous with one gene copy encoding a normal G6PD variant and one gene copy encoding a deficient G6PD variant (and a phenotype ranging from normal to deficient), or homozygous normal with both gene copies expressing G6PD variants with normal G6PD activity. Through the process of X-chromosome inactivation (also called lyonization), females express only one of their two copies of the gene in each cell. This occurs randomly in the precursors of RBCs early in the embryonic stage and results in different ratios of gene expression in mature RBCs amongst females but is constant within an individual [1]. As a consequence females heterozygous for G6PD can manifest a range of intermediate G6PD activities between typical normal and deficient G6PD activities that reflects the average enzymatic activity of these two cellular populations.

Glucose-6-phosphate dehydrogenase deficiency (G6PDd) is the most common enzymopathy with at least 400 million individuals affected worldwide [3, 4]. More than 185 clinically relevant variants of G6PDd have been reported with a spectrum of associated enzyme deficiencies [2]. The high prevalence of these mutants is likely to have been driven by their ability to provide some degree of protection from malaria [5–9]. A case control study conducted in The Gambia looking at three major polymorphisms associated with G6PD deficiency found that G6PDd provided significant protection against severe malaria (OR = 0.83) [10]. A cross sectional survey from the Brazilian Amazon reported a very strong protective effect against all species of malaria for the G6PD A-variant (OR = 0.12) and even stronger protection with the Mediterranean variant (OR = 0.01) [7]. A Thai study showed that patients with the Mahidol variant (conferring moderate G6PDd) had reduced *Plasmodium vivax,* but not *Plasmodium falciparum* parasitaemia [5]. More recently, a study conducted in Papua found detectable parasitaemia to be significantly lower among patients with G6PDd (OR = 0.44) with a greater protective effect for *P. vivax* compared to *P. falciparum* [6].

The presence of G6PDd in patients with *P. vivax* infection results in significant challenges to achieving radical cure. The only class of anti-malarial drugs currently available to eliminate the dormant liver stages of *P. vivax* (hypnozoites) are the 8-aminoquinolone compounds and these cause haemolysis in G6PDd individuals [11]. The degree of haemolysis depends on total dose of primaquine (PQ), underlying G6PD variant and age of the RBC population (with lower G6PD activities in older cells); depending on conditions, haemolysis can be slow and self-limited or result in acute and potentially fatal anaemia [11, 12]. Previous studies suggest that individuals with non-severe G6PD variants who are exposed to PQ experience an initial drop in haemoglobin (Hb) when old RBCs with low G6PD activities lyse [12]. As a response erythropoiesis increases and lysed cells are replaced by younger cells with higher G6PD activity ultimately stabilizing and reversing anaemia. Subsequent doses of PQ will not result in a further drop in Hb as the younger population of RBCs has higher G6PD activities and at the same time the rate of erythropoiesis has increased to replace lysing RBCs in due time, resulting in a so-called “resistance phase” [12]. In contrast G6PD activities in RBCs of patients with severe G6PD variants are very low even in young RBCs, therefore, sustained exposure to an oxidizing agent will result in haemolysis irrespective of the age of the RBC. Continuous treatment with PQ in these patients will result in severe and ultimately fatal anaemia. There are additional factors that contribute to risk of haemolysis at an individual level and at a specific event that are not well understood.

A number of 8-aminoquinoline compounds are under development, however to date PQ is the only drug which is licensed and widely available. Primaquine-based radical cure in G6PD normal patients is usually administered over 14 days at a total dose of either 3.5 or 7 mg/kg bodyweight depending on the assumed susceptibility of dominant local parasite strains [13]. Although either PQ regimen is usually well tolerated in G6PD normal subjects it is often not prescribed due to fears of drug-induced haemolysis; when PQ is prescribed the long treatment course, which is normally 14 days, can be associated with poor adherence and thus effectiveness [14–16]. Tafenoquine (TQ), a related 8-aminoquinoline, is at the end of phase 3 clinical trials and is anticipated to be licensed by 2018 [17–19]. The half-life of TQ is 14 days compared to 4–6 h for PQ [20], and this allows adequate cure with a single 300 mg dose [17]. A single dose regimen of TQ is likely to improve treatment adherence significantly, however may also raise the potential for sustained haemolysis in G6PDd patients and for this reason TQ is likely to be licensed for use only after G6PD normal status has been confirmed.

The current World Health Organization (WHO) malaria treatment guidelines recommend testing for G6PDd prior to administration of PQ based radical cure whenever possible [21, 22]. The gold standard for measurement of G6PD activity is UV spectrophotometry [23, 24], not suitable for field deployment or point-of-care (PoC) testing. The most widely used field test for G6PDd detection is the fluorescent spot test (FST) [25, 26], a semi-quantitative assay, which requires basic laboratory infrastructure, a cold chain, and experience for correct interpretation. A number of qualitative tests have been introduced over the last couple of years with superior operational characteristics and comparable diagnostic performance as the FST [27–30]. All of these tests have a threshold of around 30% enzyme activity, a threshold sufficient to discriminate G6PDd homozygous females and hemizygous males, from G6PD normal but inadequate for heterozygous females, with intermediate enzyme activities above 30% of normal but below a pre-defined considered safe threshold such as 70% from normal [1, 22]. The threshold of current qualitative PoCs is considered sufficient to guide PQ treatment [2], however heterozygous females are at substantial risk of drug induced haemolysis [13] and there are concerns regarding the safety of prescribing the long acting TQ in patients with enzyme activity of less than 70% [31]. Diagnosis of this higher threshold will require more discriminative quantitative tests.

Accessbio (USA) has recently developed a Biosensor™, a quantitative handheld device that measures G6PD activity based on the electrochemical properties of G6PD in blood samples [32, 33]. Other new quantitative tests are also in the development pipeline. This new class of diagnostic tools aims to address the diagnostic needs of heterozygous women as well as being flexible to different threshold activities for different 8-aminoquinoline regimens. Whilst the WHO has published recommended guidelines on the clinical evaluation of quantitative G6PD assays required for pre-qualification [34], these do not cover technical details such as sample handling, reference testing and statistical analysis. A set of recommendations and guidelines have been published earlier on the evaluation of qualitative G6PD diagnostics [24], aim of this article is to summarize suitable approaches for the field evaluation of quantitative G6PD assays.

## Challenges in the field

Field staff performing experimental G6PD testing at field level need to be appropriately trained on the experimental assay and receive training on the requirements for sample handling for later reference testing. Undertaking evaluation studies of diagnostics assays and collecting appropriate samples in malaria endemic areas can be logistically challenging. Current G6PD reference methods require significant laboratory infrastructure and well trained laboratory staff, which are lacking at many field sites. Accordingly samples collected in the field may have to be shipped to a local reference centre and this should be done in a timely manner and with a reliable cold chain.

G6PD enzyme activity and its degradation is temperature dependent, hence whole EDTA blood should not be stored at room temperature. Freezing samples and maintaining a respective cold chain in a field setting is demanding and often impossible. Whole blood samples can be stored at 4–8 °C for later analysis, however the duration at which samples can be stored and reliably assayed at a later time point is unknown. In one study conducted on 9 EDTA blood samples with normal (n = 7) and intermediate G6PD activity (n = 2) and stored at 4 °C, G6PD activity remained stable for up to 21 days with a total drop in activity of less than 5% by day 21 [35]. In contrast a study of 100 EDTA blood samples from Nigeria reported a 15–21% drop in G6PD activity after 21 days of storage at 4 °C [36]. In Malaysia, a study on 188 EDTA neonatal cord blood samples stored at 4 °C reported a minimal (0.8%) fall in G6PD activity among 172 normal individuals within the first 24 h. The decrease rose to 1.2% at 48 h, 4.7% at 4 days and 5.6% at 7 days of storage [37]. Another study from Turkey, observed G6PD activity decreasing by 11% between days 1 and 3 and 40% between days 1 and 7 when samples were stored at 4 °C [38].

Reference testing may also include flow cytometry to determine the fraction of G6PDd RBCs in heterozygous females. In these cases samples must be processed prior to shipment. Ideally samples should be cryopreserved by adding a cryopreservant such as Glycerolyte 57™ and stored at −80 °C [39]. An alternative to storing and shipping samples at very low temperatures is to replace plasma with an additive (2.5% glucose, 0.9% sodium chloride, 0.027% adenine, 0.75% mannitol) which maintains G6PD activity for longer periods than EDTA in individual cells at temperatures of 4–8 °C [35]. When the additive was added within 48 h of collection and samples were stored at 4 °C there was no significant decrease in the proportion of RBCs with high G6PD activity for up to 21 days [35]. If there is the possibility to freeze whole blood samples at −80 °C and ship on dry ice, this may also be done, however RBCs will lyse during thawing. The consequences of this lysis are several fold: (1) specimen integrity is compromised, (2) the G6PD enzyme is less stable in lysed blood than in intact RBCs and the samples should be used within 1 h of thawing and kept on ice during that period of time, and (3) the evaluation cannot include an assessment of RBC lysis (as this has already occurred in the freeze-thaw process) but only enzyme activity.

In most field studies experimental point of care tests (PoCs) are likely to be tested directly on a finger prick using capillary blood, whereas reference testing is more likely to be undertaken on venous samples; this has the potential to introduce a bias [40]. A study in Thailand assessed relevant blood parameters on 139 paired capillary and venous blood samples and found significantly lower values for red blood cell count, haemoglobin concentration, haematocrit, mean corpuscular volume and significantly higher values in platelet count in capillary compared to venous blood; with the exception of the platelet count the difference was less than 5% for all parameters [40]. The G6PD activity of paired samples collected from venous and capillary sampling were similar when measured by spectrophotometry (Trinity, UK) (median 7.51 and 7.61 U/gHb, respectively) [40]. Similarly, an evaluation of qualitative tests by FST and Carestart G6PD RDT (Accessbio, USA) showed 100% concordance between paired capillary and venous samples [41].

## Reference method and universal cut off activities

The evaluation of a novel quantitative assay requires comparison with an appropriate quantitative reference method. Additional tests such as genotyping and flow cytometry provide complementary information on underlying G6PD variants and heterozygosity. Several quantitative assays have emerged that either directly or indirectly measure NADPH formation as a result of G6PD activity [2], however the majority of test evaluations have used spectrophotometry as the reference method. Spectrophotometry measures the formation of NADPH from G6PD activity by measuring the difference in absorbance (Δ absorbance) of the sample at 340 nm over time [42] and requires a kinetic and temperature controlled spectrophotometer, trained laboratory staff and a well-functioning laboratory infrastructure. Spectrophotometry measurements are usually run in duplicate, and the inclusion of at least commercial G6PD deficient and G6PD normal controls is highly desirable. Prior to being considered as reference method spectrophotometry must be fully validated, including performance of operators, reproducibility and internal as well as external quality assurance procedures must have been established.

The WST8/1-methoxy PMS test is a robust and cost effective alternative to estimate G6PD activity that can be performed on dried blood spots and whole blood samples. The WST8/1 test is based on the formation of an orange formazan dye generated by G6PD enzyme activity and facilitated by the 1-methoxy PMS as an electron carrier [43]. The assay has been adapted for quantitative batch testing in a 96 well microplate and can be read by ELISA reader [44, 45]. A preliminary field evaluation showed discrepancies between spectrophotometry and the methoxy test, although the assays were not run simultaneously [32], additional evaluation studies are under way.

Quantification of G6PD activity requires normalization of the result to account for RBC mass and the temperature of the assay. Whenever possible an RBC count is preferable to Hb measurement, since the low Hb concentrations in anaemic patients may result in an artificial increase of G6PD activity, whereas this is not the case when the RBC count is used for normalization [42, 46].

Absolute values can vary between different assays [23] and hence results need to be transformed into population specific values relative to the population specific adjusted male median (see below) [24]. Presentation of the results without the corresponding adjusted male median (AMM) value significantly confounds the comparison of the assay results across different studies. Even then performance criteria are likely to differ if different reference assays are used. To the extent possible a single reference assay should be used [24].

To date no universal cut-off and corresponding method that defines G6PDd has been clearly adopted. The degree of G6PD activity can help to guide clinical management, but the relevant threshold varies with the intended use. For example the threshold currently used to guide PQ radical cure is 30% of the AMM [2], whereas the proposed threshold activity for TQ treatment will be approximately 70–80% to ensure safe use in females with intermediate activity [22, 31]. The great difference in absolute values measured among different spectrophotometric assays does not permit the definition of the optimal threshold if different assays are used. The problem is well illustrated by the package insert of a standard G6PD normal control supplied by Trinity Biotech (Cat. No. G6888) which provides an assigned activity range of 12.6–23.4 U/gHb. Although it would be highly desirable to use a standard that provides a constant Δ absorbance with which to normalize results, to date none has been produced.

## Sample size and statistical analysis of results

In order to achieve reliable results, all studies need a sample size sufficient to ensure precise and reliable results, but small enough to be feasible in an endemic setting. Unless the aim of an evaluation study is WHO prequalification where static specific sample sizes are required (see below) [34], the sample size can be adjusted to reflect the local epidemiology and demographics of the population. There are numerous sample size calculators available online [47–50] that can perform sample size calculations, as well as a range of statistical software packages. Three approaches are generally considered to calculate the sample size for evaluating novel quantitative G6PD diagnostics.

If the intended setting and use of the novel diagnostic is known, a decision on whether specificity or sensitivity is of greater interest can be made and the most relevant threshold activity defined. Following these considerations sample size can be calculated based on the assumed performance of the novel diagnostic at a specific threshold activity following recommendations from the special programme for research and training in tropical diseases (TDR) [51].

In evaluating G6PD tests the sample size is driven primarily by the prevalence of G6PD deficiency which will determine the number of people that need to be screened to achieve the desired positives (G6PDd) for the desired confidence interval and power. A more general approach is to calculate the sample size for a two sided, paired *t* test, after stating the minimal clinically relevant difference between the mean results of the novel assay and reference method, detectable at a given alpha value (likelihood that H_0_ is erroneously rejected; usually set at 95%) and power (likelihood that H_0_ is correctly rejected; usually set at 80%). Sample size can then be calculated according to procedures outlined by Dell et al. [52]. In many cases the absolute difference between gold standard and experimental assay is less relevant, than the correlation between the two measures. Hence a third approach is to determine the minimal correlation coefficient between gold standard and experimental assay to be detected and calculate sample size accordingly [53, 54]. Irrespective of the method applied the calculated sample size should be increased by 10–20% to accommodate for losses due to errors in study procedures and other problems.

**Fig. 1:**
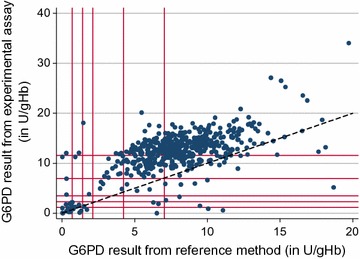
Example of a scatter plot with line of equality. Starting from origin, red vertical and horizontal lines correspond to 10, 20, 30, 60 and 100% G6PD activity of the adjusted male median. The black dotted line = line of equality

Since different quantitative assays can have different absolute values [23], a direct comparison of results of the experimental assay and reference method is not informative. The most immediate comparison that can be made between the new G6PD test and the gold standard is a direct correlation of results and calculation of the correlation coefficient. Plotting results of the experimental assay and reference method in a scatter plot including a line of equality is a simple and clear way to display the relation between experimental and reference method test results (Fig. 1). This can be augmented by the inclusion of thresholds for 10, 30, 60 and 80% enzyme activity to show the relative performance of the assay at low, intermediate and high G6PD activity.

**Fig. 2:**
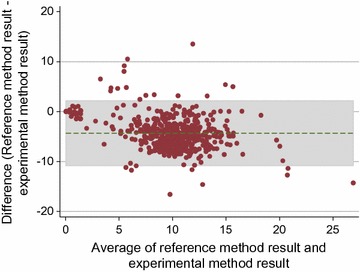
Example of a Bland–Altman plot. Grey shaded areas indicate 95% LoA, green dotted line = mean difference, difference between 0 and the mean difference = mean bias

Absolute quantitative values can be compared using Bland and Altman plots which show the average of two paired results on the x-axis against the difference of the same results on the y-axis [55] (Fig. 2). From this plot the mean difference between both assays and a 95% limit of agreement (LoA) (mean difference ± 1.96 standard deviations) can be calculated. The standard deviations should also lie within clinically acceptable ranges of difference in G6PD activity. A mean difference deviating from zero suggests a systematic difference between absolute readings of experimental and reference method, and potentially a systematic error in the experimental assay. The relevance of the detected mean difference is dependent on the intended use of the experimental assay. The clinical relevance of any difference and LoA is made by the investigator and is guided by practical considerations.

**Fig. 3:**
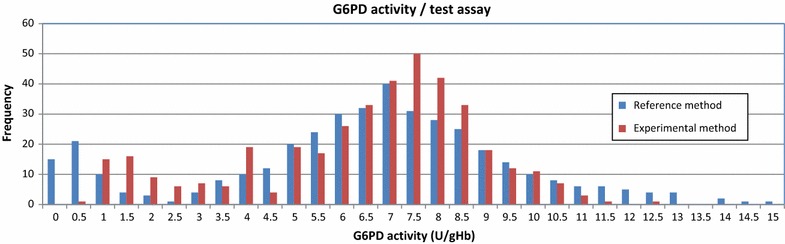
Example of a histogram to show G6PD activity distribution/test assay

**Fig. 4:**
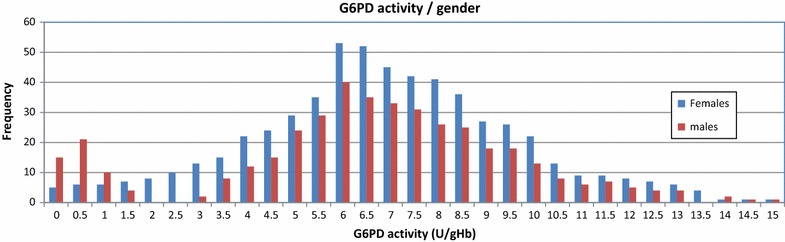
Example of a histogram to show G6PD activity distribution/gender

A histogram of G6PD activity, stratified by gender within a study population is a simple way to present the distribution of G6PD activity within a population. A respective histogram allows visual representation of the absolute values of different assays and whether an assay underdiagnoses samples beyond a specific G6PD cut-off activity. Within a non-skewed population of sufficient size the majority of individuals will have G6PD activities normally distributed around a population specific median. A second smaller peak at the low end of the G6PD activity axis represents G6PDd individuals. Individuals in between these two peaks are intermediate G6PD deficient and will mostly consist of heterozygous females. Major differences in the form of the G6PD activity distribution of experimental assay and reference method are an indicator of short comings of the experimental assay (Fig. 3), a non-bimodal distribution of results among the male population further indicates short comings in the underlying method (Fig. 4).

**Fig. 5:**
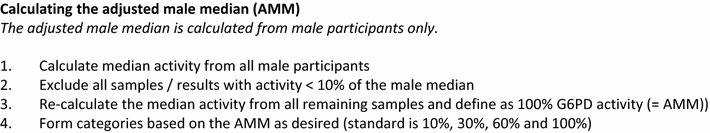
Calculating the adjusted male median (AMM)

Defining assay specific absolute values representing 100% G6PD activity allows measured quantitative G6PD results to be categorized which in turn enables performance indicators to be calculated at various cut-off activities [24, 56]. The current standard approach for the definition of a study population specific 100% G6PD activity is to calculate the adjusted male median (AMM) (Fig. 5) [24], alternatively if results from genotyping are available at time point of analysis then the median activity of all wild type participants (wt) can be calculated [40]. Cut-off activities for categories of G6PD activity can then be defined as fractions of the AMM or median of all wt as required [24, 27, 57] (Fig. 1). In order to assess the threshold activity at which the experimental assay performs best, results of the reference method are transformed into a dichotomous outcome according to defined cut-off activities. Calculating areas under the reactor operating curve (ROC) allow for a direct comparison of performance of the experimental assay at different cut-off activities [58]. However, this procedure is unnecessary for experimental assays intended for a specific use such as guiding PQ treatment, where a desired threshold activity is already known.

Once appropriate threshold activities have been established, test results of experimental assay and reference method can be categorized into true negative, true positive as well as false positive and false negative results to calculate standard performance indicators [51]. In this context it is desirable to change the nomenclature to avoid confusion and refer to test results as G6PD deficient rather than positive and G6PD normal rather than negative results. Results of this analysis can be summarized in an extended 2 × 2 table (see Table 2 in [32]).

## WHO recommendations to establish clinical performance for assay pre-qualification

Following field evaluation studies, successful quantitative G6PD diagnostics may undergo procedures for prequalification with the WHO. The WHO has published a guideline for WHO prequalification of in vitro diagnostic medical devices (IVDs) to identify G6PD activity [34]. The guideline provides “technical guidance to in vitro diagnostic medical device (IVD) manufacturers that intend to seek WHO prequalification of IVDs for the detection of glucose-6-phosphate dehydrogenase (G6PD) deficiency” [34], however does not list procedures to demonstrate clinical utility.

## Discussion and outlook

This review presents a number of recommendations to facilitate standardized evaluation of quantitative G6PD activity test assays (Table 1). Standardized evaluation studies are necessary to provide a comparable basis for subsequent pooled analyses. As novel quantitative G6PD diagnostics become available their potential to provide robust quantitative G6PD results suitable for field deployment needs to be assessed. The most recent generation of instruments contain an integrated temperature correction factor and Hb measurement and G6PD activity is displayed normalized as U/gHb. Some have argued that a quantitative G6PD result is too complex for translation into treatment at field level [59]. However, if universal cut-off activities can be established, training materials can be developed to link quantitative values to treatment decisions.

**Table 1:** Overview of recommendations for field evaluation of quantitative G6PD assays

Task	Recommendation	Reference
Sample size for performance evaluation	Two approaches, based on: • Desired performance indicators • Minimal difference in means measured by two methods	[51, 52]
Sample size WHO pre-qualification	• 200 G6PD specimens • 200 G6PD intermediate specimens • 1000 G6PD normal specimens	[34]
Sample	Venous or capillary blood	[40, 41]
Max time to reference testing	4–7 days	[35–38]
Sample storage temperature	At 4–8 °C	[35–38]
Reference testing	Spectrophotometry	[24, 42]
Statistical methods	• Bland–Altman plot • Scatter plot • Histogram of G6PD activity distribution/test assay • ROC analysis • Adjusted male median • Sensitivity, specificity, positive and negative predictive value	[24, 55, 56]

Although current biosensors are not as precise as spectrophotometry [32], performance is likely to improve and their versatility and ability to discriminate individuals with intermediate enzyme activity has the potential to provide a convenient platform to facilitate G6PD testing and TQ use across different endemic settings.
